# Investigation of the Metabolic Profile and Toxigenic Variability of Fungal Species Occurring in Fermented Foods and Beverage from Nigeria and South Africa Using UPLC-MS/MS

**DOI:** 10.3390/toxins11020085

**Published:** 2019-02-01

**Authors:** Ifeoluwa Adekoya, Patrick Njobeh, Adewale Obadina, Sofie Landschoot, Kris Audenaert, Sheila Okoth, Marthe De Boevre, Sarah De Saeger

**Affiliations:** 1 Department of Biotechnology and Food Technology, University of Johannesburg, Doornfontein 2092, South Africa; pnjobeh@uj.ac.za; 2 Department of Food Science and Technology, Federal University of Agriculture, PMB, 2240 Abeokuta, Nigeria; obadinaw@gmail.com; 3 Department of Plants and Crops, Faculty of Bioscience Engineering, Ghent University, Valentin Vaerwyckweg 1, B-9000 Ghent, Belgium; Sofie.Landschoot@ugent.be; 4 Laboratory of Applied Mycology and Phenomics, Department of Plants and Crops, Ghent University, B-9000 Ghent, Belgium; Kris.Audenaert@ugent.be; 5 Department of Botany, School of Biological Sciences, University of Nairobi, P.O. Box, Nairobi 30197, Kenya; dorisokoth@yahoo.com; 6 Centre of Excellence in Mycotoxicology and Public Health, Ghent University, B-9000 Ghent, Belgium; Marthe.DeBoevre@ugent.be (M.D.B.); Sarah.Desaeger@ugent.be (S.D.S.)

**Keywords:** toxigenicity, *Aspergillus*, mycotoxins, metabolites, UPLC-MS/MS, fermented foods

## Abstract

Fungal species recovered from fermented foods and beverage from Nigeria and South Africa were studied to establish their toxigenic potential in producing an array of secondary metabolites including mycotoxins (*n* = 49) that could compromise human and animal safety. In total, 385 fungal isolates were grown on solidified yeast extract sucrose agar. Their metabolites were extracted and analyzed via ultra-performance liquid chromatography tandem mass spectrometry. To examine the grouping of isolates and co-occurrence of metabolites, hierarchal clustering and pairwise association analysis was performed. Of the 385 fungal strains tested, over 41% were toxigenic producing different mycotoxins. *A. flavus* and *A. parasiticus* strains were the principal producers of aflatoxin B_1_ (27–7406 µg/kg). Aflatoxin B_1_ and cyclopiazonic acid had a positive association. Ochratoxin A was produced by 67% of the *A. niger* strains in the range of 28–1302 µg/kg. The sterigmatocystin producers found were *A. versicolor* (*n* = 12), *A. amstelodami* (*n* = 4), and *A. sydowii* (*n* = 6). Apart from *P. chrysogenum,* none of the *Penicillium* spp. produced roquefortine C. Amongst the *Fusarium* strains tested, *F. verticillioides* produced fumonisin B_1_ (range: 77–218 µg/kg) meanwhile low levels of deoxynivalenol were observed. The production of multiple metabolites by single fungal species was also evident.

## 1. Introduction

Secondary metabolism in filamentous fungi facilitates the synthesis of various chemical compounds including mycotoxins, which are unnecessary for normal development and growth. A number of them have been widely exploited for various biological applications as anti-infective, antibiotics, and anticancer agents to name a few [1]. On the other hand, some secondary metabolites are mycotoxins, which are poisonous substances that have deleterious impact on the health of animals and man. Aside from their secondary metabolism, filamentous fungi (e.g., *Aspergillus niger, A. awamori*, and *F. oxysporum*) play important role in enzyme production while others like *A. luchuensis* and *A. oryzae*, and are useful in food fermentations, [2] being atoxigenic.

Notwithstanding in the case of fermented foods, an important factor is the safety of the indigenous and competing microbial species/strains due to possible synthesis of poisonous secondary metabolites including mycotoxins. Previous works [3,4,5] showed the high occurrence of potentially mycotoxigenic fungi in fermented products namely fermented: African oil bean seed (*ugba*), sorghum gruel (*ogi baba*), locust beans (*iru*), maize gruel (*ogi*), maize meal (*mahewu*), melon (*ogiri*), and cereal based opaque beer (*umqombothi*) from Nigeria and South Africa and it was expedient to determine the toxigenic profile of the fungi present in these products which this study addressed. *Ogi*, *umqombothi*, and *mahewu* are obtained primarily from maize through lactic acid fermentation. *Ogi baba* is also a product of similar fermentation process with *ogi* but has different substrate (sorghum), both are consumed amongst children and adults mainly in West Africa while the consumption of *mahewu* and *umqombothi* is predominately amongst black citizens in South Africa. Furthermore, *ogiri*, *ugba*, and *iru* are alkaline fermented vegetable protein that outstanding numbers of West Africans rely on to meet their needs for calories, protein, and vitamins as low-cost meat substitutes and condiments [6]. It is undebatable that fungal contamination in these products heightens the risk of mycotoxin contamination.

Aflatoxins (AFs) are produced amongst members of the *Aspergillus* genera, particularly *A. parasiticus* and *A. flavus* [7]. Aflatoxins are considered the most toxic mycotoxins and this group includes AFB_1_ which is a Group 1 carcinogen according to the International Agency for Research on Cancer (IARC) [8]. During AFs biosynthesis, various metabolites are synthesized as precursors that have much lesser toxicity e.g., O-methylsterigmatocystin (OMST) and versicolorins [9,10]. Kojic acid (KA), cyclopiazonic acid (CPA), ochratoxin A (OTA), and patulin (PAT) are examples of poisonous secondary metabolites produced by *Aspergillus* species.

One of the hepatotoxic and nephrotoxic mycotoxins is OTA, which is mainly produced by *A. ochraceus* in the tropics and by *P. verrucosum* in warm climates, *A. niger* also produce OTA. Within the *Fusarium* genus, producers of fumonisins (FBs), trichothecenes (TCs), and zearalenone (ZEN) have drawn the highest recognition [11]. For example, *F. oxysporum F. verticillioides*, and, *F. proliferatum* are examples of FB producers [12] while *F. graminearum* and *F. culmorum* are prominent deoxynivalenol (DON) producers [13]. *Fusarium* mycotoxins exhibit some estrogenic immuno-suppressive and mutagenic effects in animals and humans [11]. The resulting impact of toxin producers on health makes it expedient to study the toxigenic properties of fungi commonly found in foods. Although mycotoxins production is constricted to few fungal strains or species, multiple toxin synthesis by a single strain or specie also exists. In addition, the toxin-synthesizing capability of mycotoxigenic fungi is impacted by varying environmental and growth factors facilitating the production of different mycotoxins singly or simultaneously [14].

Currently, the polyphasic taxonomic technique which entails physiological, morphological, and biochemical characterization e.g., using DNA sequence analysis and ultra-performance liquid chromatography tandem mass spectrometry (UPLC-MS/MS) is progressively being utilized for fungal groupings a result of their complex taxonomy [15]. However, numerous secondary metabolites have been inadequately studied [16], multiple toxin production have also not been fully elucidated, but resultant synergistic effects have been demonstrated [14,17]. According to Aldars-Garcia et al. [18], understanding the variability amongst fungal isolates and their interaction needs to be considered in developing measures to govern growth and predicting associated risks. It is therefore paramount to establish the toxigenic potentials of various *Penicillium*, *Aspergillus*, and *Fusarium* spp. isolated from different fermented products, which have not been previously studied. To achieve this objective, several isolates of *Penicillium*, *Aspergillus*, and *Fusarium* spp. previously obtained from seven different fermented products from Nigeria and South Africa, after confirming their identities by molecular means [3,4,5] were evaluated for their capability in producing mycotoxins and other secondary metabolites. In this regard, a diversity of secondary metabolites was analyzed via UPLC-MS/MS. 

## 2. Results and Discussion

Table 1 and Table 2 show the mycotoxins produced by the *Aspergillus*, *Penicillium* and *Fusarium* spp. recovered from the fermented products. *Aspergillus* spp. (*n* = 240) were screened for 34 secondary metabolites synthesized by various *Aspergillus* spp. as reported by Samson and Varga [7], Larsen et al. [19], and Samson et al. [20]. Generally, species within the *Aspergillus* section *Flavi* exhibit close phylogenetic connections and morphology, and are classified as domesticated or aflatoxigenic species [21]. *Aspergillus parasiticus* and *A. flavus* are the principal aflatoxigenic species that are linked with AFs contamination of agricultural commodities. In our study, up to 70% of *A. flavus* isolates produced AFB_1_ (range: 27–4406 µg/kg), while 77, 64, 31, and 19% of *A. parasiticus* isolates (*n* = 36), respectively, produced AFB_1_ (range: 89–3602 µg/kg), AFB_2_ (range: 37–566 µg/kg), AFG_1_ (range: 36–322 µg/kg), and AFG_2_ (range: 34–664 µg/kg), revealing that the *A. parasiticus* isolates had different capabilities in producing the four AFs. The variability in the metabolic profile of the *Aspergillus* isolates might be associated with genetic recombination [22,23]. For example, Okoth et al. [23] reported the production of both AFB’s and AFG’s by *A. flavus* isolates in their study, which is at variance with the traditional chemotypes of the analogous species.

**Figure 1:**
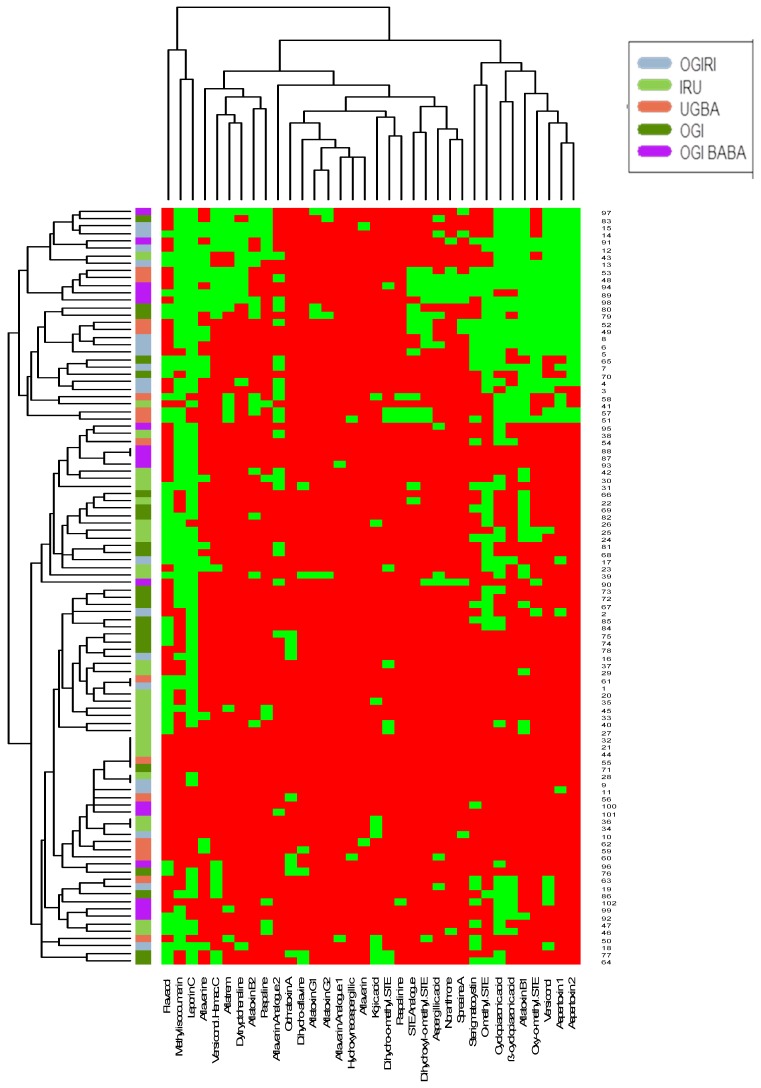
Hierarchical grouping established from the metabolite profile of *Aspergillus* species recovered from fermented foods from Nigeria. Color codes reveal the absence (red) or presence (green) of particular toxin or metabolite. Numbers listed corresponds with isolated species listed in the Supplementary File.

**Figure 2:**
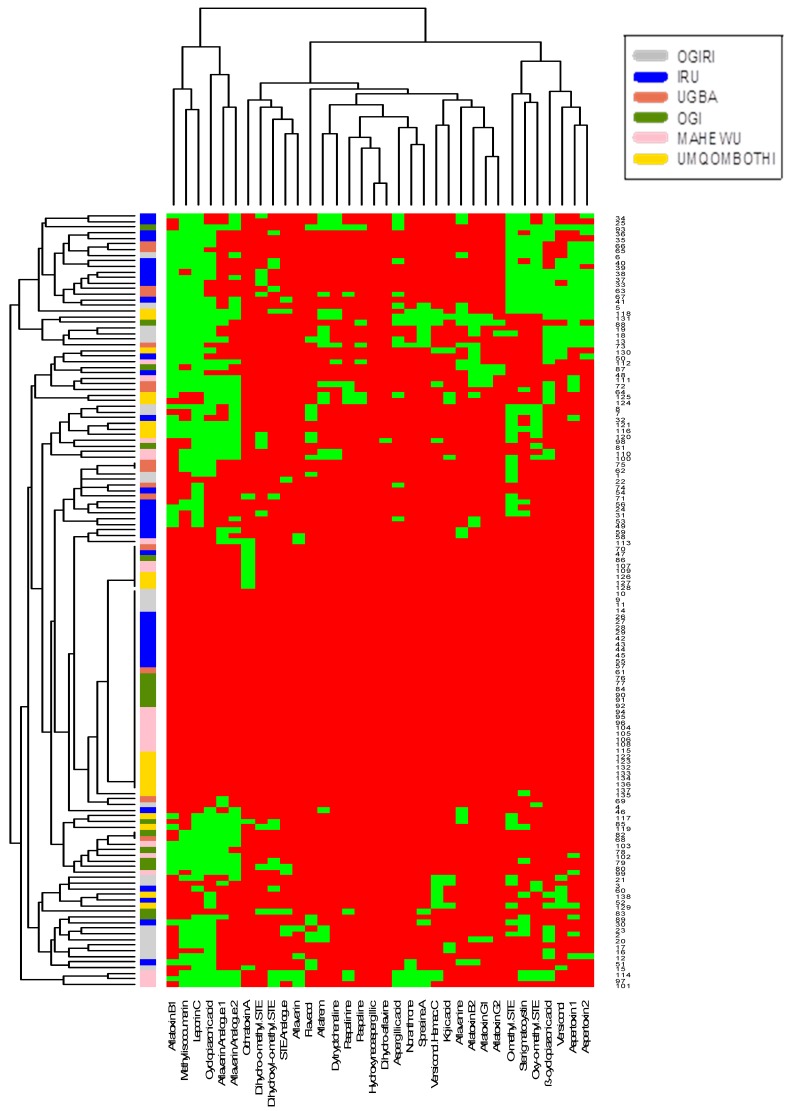
Hierarchical grouping established from the metabolite profile of *Aspergillus* species recovered from fermented foods from South Africa.

*Aspergillus oryzae* belongs to the domesticated group with *A. sojae* and *A. tamarii*, which are commonly used for fermenting foods [21]. This is in line with our research wherein AFs synthesis was not observed among the *A. oryzae* strains present (*n* = 2) in the fermented food (*ogiri*), but some of its extrolites were found, particularly KA and CPA. The most commonly used extrolites in species identification are CPA, AFs, aspergillic acid (AA), and KA [7,24], and each species is usually distinguished by a particular metabolic profile [19] but some might not synthesize the expected metabolites [21,25]. For example, *A. parasiticus* is not affiliated with CPA synthesis and is differentiated from *A. flavus* based on this. Another group of *A. flavus*-closely linked isolates—*A. minisclerotigenes* produced AFB_1_ (range: 96–242 µg/kg), AA, aflavarin (AFV), aflatrem (AFTR), KA, CPA, paspaline (PAS), and aflavinine (AFN) (Figure 1 and Figure 2).

**Figure 3:**
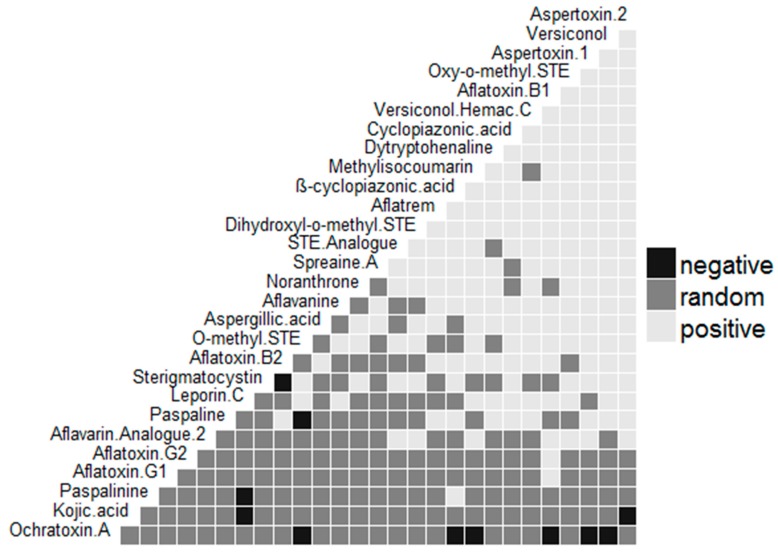
Co-occurrence matrix of metabolites of *Aspergillus* species recovered from fermented foods from Nigeria. A positive association illustrates that with the occurrence of a particular metabolite, its most likely that the other metabolite will be present. A negative association illustrate that with the occurrence of a particular metabolite, it is most likely that the other metabolite will not occur. A random association illustrates that nothing can be said about the co-occurring metabolites.

**Figure 4:**
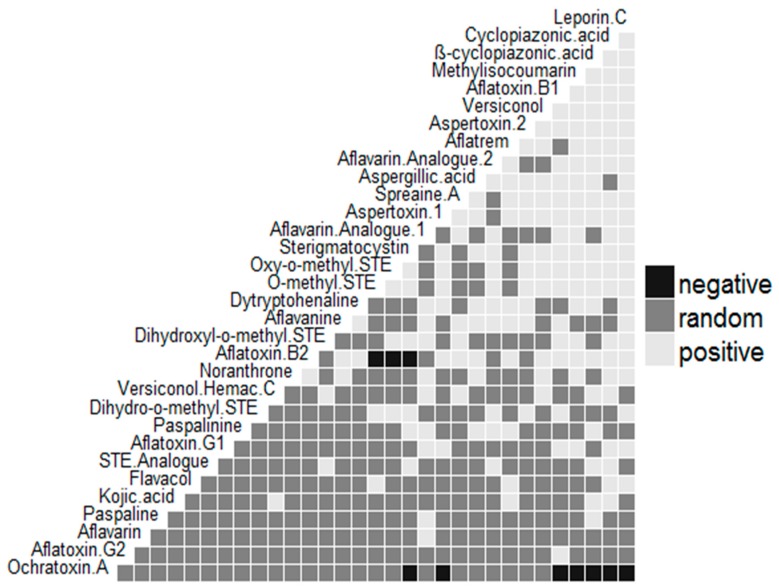
Co-occurrence matrix of metabolites of *Aspergillus* species recovered from fermented foods from South Africa.

Figure 1 and Figure 2 further show the hierarchical grouping based on metabolite profiles of the *Aspergillus* spp. recovered from the foods represented as heat maps wherein the presence of a particular metabolite was shown as green and its absence as red. The dendrogram revealed sterigmatocystin (STE), AFB_1_, versiconol (VOH), OMST, aspertoxin 1 (ASPT), and aspertoxin 2 (ASPT-2) (Figure 1), and OMST, STE, ß-CPA, ASPT, VOH, and ASPT-2 (Figure 2) as major compounds for chemical clustering. These metabolites have been reported to be AF precursors within their biosynthetic pathways [9,10], whereas ASPT is the 12c-hydroxy derivative of OMST, a precursor of AFB_1_ and AFG_1_ [26]. As revealed in Figure 1, isolates that had similar origins coincided in *iru*, but this is not always so, which point to the fact that isolates that have the same origin do not always possess comparable metabolic profiles [27]. Interestingly, AFB_1_ and CPA had a positive pairwise association (Figure 3 and Figure 4), which denotes that if AFB_1_ is detected, there is a high probability that CPA will also be present or vice versa. This conforms to the findings of Vaamonde et al. [25] on the co-contamination of agricultural commodities by AFB_1_ and CPA. In a study by Zorzete et al. [28], co-occurrence of AFB_1_ and CPA was observed in 11% of 70 peanut kernel samples from the analyzed cultivars.

According to Mogensen et al. [29], up to 75% of *A*. *niger* isolates produced FB_2_ and 41% produced OTA. Based on our findings, among the 24 species of *A*. *niger* tested, 67% produced OTA (range: 28–1302 µg/kg), while none produced FB. Same as in our study, Larsen et al. [15] did not observe the concurrent production of FB and OTA by *A. niger*, although the production of both mycotoxins is strain dependent [30]. Consequentially, the production of OTA by *A*. *tubingensis* has been overrated in some research based on the provision of false positives established through HPLC fluorescence detection [31]. OTA was not produced by *A*. *tubingensis* as we observed as previously confirmed by [15,16]. *A. sclerotium* within the *Aspergillus* section *Circumdati* confer some ochratoxigenic potential [7], but only 20% of the isolates tested (*n* = 5) produced OTA (161 µg/kg). STE has been linked with genotoxic and teratogenic effects in experimental animals and accepted as a potential carcinogen [32]; STE was produced by 25% of *A. amstelodami,* 75% of *A. versicolor,* and 50% of *A. sydowii*. 

*Aspergillus* metabolites production by *A. ruber*, *A. candidus*, *A. clavatus*, *A. tritici,* and *A. ustus* isolates was not observed. Knowledge of the biochemistry of these fungal species still needs to be explored. None of the *Penicillium* spp. were toxin (roquefortine C [ROQ C] and OTA) producers except *P. chrysogenum* and *P. verrucosom*. Only six *P. verrucosum* strains were present and 67% of them produced OTA within the range of 15 and 320 µg/kg whereas ROQ C was produced by 41% of the *P. chrysogenum* strains (*n* = 29, range: 13–1260 µg/kg). 

The commonly occurring *Fusarium* mycotoxins are FB_1_, FB_2_, FB_3_, DON, T-2 toxin (T-2), diacetoxyscirpenol (DAS), zearalenone (ZEN), 3-acetyldeoxynivalenol (3-AcDON), neosolaniol (NEO), 15-acetyldeoxynivalenol (15-AcDON), HT-2 toxin (HT-2), fusarenon-X (FUS-X), nivalenol (NIV), and fusaric acid (FA) [33]. The mycotoxins produced by 38, 14 and 24% of *F. verticillioides* (*n* = 21) were FB_1_ (range: 77–218 µg/kg), FB_2_ (range: 63–234 µg/kg) and FB_3_ (range: 79–205 µg/kg), respectively. As observed in this study, the fungal species that is mostly associated with FB production is *F. verticilliodes* [12] and their FB producing abilities were lesser compared to some studies [14,34] and relatively lower range of FB_1_, FB_2_ and FB_3_ were produced on the yeast extract sucrose media. In the study of Shi et al [14], *F. verticillioides* produced FB_1_ with average concentrations of 15,168 µg/kg in PDA, 273,894 µg/kg in rice and 237,208 µg/kg in maize medium, respectively. Since, mycotoxigenic potential of fungi is largely dependent on isolated hosts, conditions of inoculation such as composition of media, or physical factors like pH, temperature, water availability, nutrient availability etc. [34,35], and these factors might have been responsible for the levels of FB observed. Subsequent studies should therefore put the optimization of these factors into consideration.

*F. fujikuroi* also produces FBs, but this was not established in our study, and its mycotoxigenic potential has also been highlighted to be extensively dependent on the conditions of inoculation [34]. Type A TCs (DAS, HT-2, NEO and T-2) were produced by *F. sporotrichioides,* whereas the maximum concentration of T-2 produced was 1,749 µg/kg. Type A TCs are severely potent in mammals with T-2 having 10 times potency compared to DON [36]. In general, a low incidence of DON was observed among the *Fusarium* isolates. According to Frisvad et al. [37] secondary metabolite profiling and production is a reliable way of distinguishing fungal species. This was helpful during our study as *Fusarium* spp. present in *ugba* and *iru* that were impossible to identify indicated a close relationship with *F. graminearum* based on their ability to synthesize DON, ZEN, and 3-AcDON but this needs to be further confirmed. 

The toxigenic potential of the fungi isolated from the fermented foods could be as a result of the food matrices. Vaamonde et al. [25] observed variation in AFs produced by strains of *Aspergillus* section *Flavi* recovered from peanut, wheat, and soybean. Whereas similar microbiota were observed among the fermented foods from Nigeria and South Africa, and there were variations in the ratio of positive species that produced low, intermediate, and heightened levels of mycotoxins. Geographical location also influences mycotoxin production amongst fungal species or strain [15]. Higher prevalence of toxigenic *A. flavus* strains was observed by Okoth et al. [38] compared to non-toxigenic strains in five out of the six locations in Kenya. Specifically, 42% of all the isolated fungi (*n* = 385) were toxigenic and comparatively, a higher occurrence of toxigenic strains was observed amongst fungal species isolated from fermented foods obtained from Nigeria (47%, *n* = 81/175) when compared to those isolated from fermented foods obtained from South Africa (39%, *n* = 78/200). Although, the inherent climatic conditions that prevail in Nigeria might have favored the observed higher toxigenic potential of isolates, emphasizing a higher risk of mycotoxin contamination and exposure amongst Nigerian consumers as opposed to South African consumers

Bankole and Adebanjo [39] related the elevated mycotoxin contamination levels in Nigerian foods to the presence of toxigenic fungi and previous [3,4] and present studies strengthens this. However, it is worthy to note that the mycotoxigenic potential of these fungi does not necessarily imply that the fermented foods contain or will contain these mycotoxins particularly at the reported levels but still it exasperates a risk. For example, *A. parasiticus* isolated from *iru* produced AFB_1_ (range: 206–445 µg/kg), AFB_2_ (36–59 µg/kg), AFG_2_ (49–71 µg/kg), and *F. sporotrichioides* produced T-2 (139–1749 µg/kg), whereas when the same *iru* sample was analyzed for these mycotoxins, AFB_1_ ranged from 3–7 µg/kg, AFG_1_, AFG_2_, and T-2 were absent [5] and similar variation was observed in other fermented products [4,5,6,40]. Accordingly, this is the first study in both countries to evaluate the potential toxigenic capacity of *Aspergillus*, *Fusarium*, and *Penicillium* spp. isolated from fermented foods.

## 3. Conclusions

The diversity in secondary metabolites including mycotoxins from *Aspergillus*, *Fusarium*, and *Penicillium* isolates evaluated using UPLC-MS/MS divulged a wide array of metabolites produced by 385 different fungal species that contaminated fermented foods from Nigerian and South African markets. The presence of principal mycotoxins (AFB_1_, STE, DON, FB_1,_ ROQ C, OTA, and ZEN) and some other toxic metabolites (KA, CPA, and VHA) was established. We delineated that toxigenic fungal species were lower than non-toxigenic species in terms of occurrence, but the levels of mycotoxins synthesized by some of the species were rather too high. The potential to produce multiple metabolites by a single fungal species or several of them under the same condition was established. The study formed a basis to further investigate and establish the degree with which these fermented foods were contaminated by mycotoxins. Nonetheless, the toxic effects of the levels of metabolites recovered need to be investigated further. This study is therefore critical towards enhancing mycotoxin monitoring, and management so as to ensure public health and safety. 

## 4. Materials and Methods

### 4.1. Reagents and Standards

Glacial acetic acid and LC-MS grade methanol were procured from Biosolve B.V. (Valkenswaard, Netherlands). Dichloromethane (DCM), ethyl acetate, acetonitrile (ACN), and ammonium acetate (analytical grade) were purchased from Merck (Darmstadt, Germany; Johannesburg, South Africa). A Millipore Milli-Q gradient system (Brussels, Belgium) was utilized for water purification. 

### 4.2. Standards

Standards used for the quantification of *Aspergillus* metabolites included AFB_1_, AFB_2_, AFG_1_, AFG_2_, OTA, and STE, which were acquired from Sigma-Aldrich (Bornem, Belgium). Standards of other *Aspergillus* metabolites listed in Table 3 were not available hence, they were qualitatively determined. Standards for the quantification of *Fusarium* metabolites including T-2 toxin (T-2) and DAS were procured from Biopure Referenzsubstanzen (Tulln, Austria). Zearalenone, DON, FB_1_, FB_2_, 3-AcDON, 15-AcDON, NEO, NIV, HT-2, and FUS-X were purchased from Sigma-Aldrich (Bornem, Belgium). FB_3_ was purchased from South African Medical Research Council (Tygerberg, South Africa).

### 4.3. Origin of Fermented Food Samples

Between February 2015 and July 2016, fermented products were collected via cluster sampling from markets located in southwestern Nigeria, and the Tshwane (25.6051° S, 28.3929° E) and Johannesburg (26.2041° S, 28.0473° E) municipalities in Gauteng, South Africa. A total of 399 samples were obtained from both locations in southwestern Nigeria (*ogi* = 35, *ogi baba* = 35, *iru* = 60, *ugba* = 30 and *ogiri* = 31) and Gauteng, South Africa (*ogi* = 33, *mahewu* = 21, *ugba* = 25, *iru* = 66, *ogiri* = 31 and *umqombothi* = 32). The fungal load and microbiota of the fermented foods was evaluated via isolation, microscopic, macroscopic and molecular characterization as described by Adekoya et al. [3]. A diversity of fungal specie were identified (*n* = 804) including potentially toxigenic *Aspergilli* (240), *Penicillia* (96), *Fusaria* species (49) and species belonging to other fungal genera (419) which did not produce typical mycotoxins. 

### 4.4. Sample Preparation

The ability of the *Aspergillus* (240), *Penicillium* (96), and *Fusarium* (49) species previously recovered and identified from the fermented products to synthesize secondary metabolites was investigated. Unmixed cultures of identified isolates were sub-cultured unto petri dishes with solidified yeast extract sucrose (YES) agar (yeast extract powder (20 g) and sucrose (100 g) were dissolved in 1 L of sterile distilled H_2_O and autoclaved at 121 °C for 15 min) using the streak plate technique, and the plates were incubated at 25 °C for 3 weeks.

### 4.5. Multi-Mycotoxin Extraction

*Aspergillus* and *Penicillium* metabolites were extracted from 336 solid cultures using the agar plug technique [12]. Unmixed fungal colonies and media (1 g) were plugged into an amber vial containing 1 mL HPLC grade methanol using a 9 mm cork borer. The content was vortexed (1 min), filtered via a 0.2 µm Millex syringe filter unit (Merck, Darmstadt, Germany) into another amber vial and the filtrate was dried with nitrogen gas. The dried extracts were reconstituted in 1 mL of injection solvent consisting of 60:40 (*v/v*) of mobile phase A and B (A: 5 mM ammonium acetate, water/methanol (95/5, *v/v*), and 0.1% formic acid; B: 5 mM ammonium acetate, methanol/water (95/5, *v/v*), and 0.1% formic acid) and vortexed for 1 min. The extract was dispensed into Ultrafree® PVDF centrifugal filters (Merck, Darmstadt, Germany), centrifuged at 14,000× *g* for 5 min, transferred into vials and subjected to analysis. 

For *Fusarium* toxins, they were extracted from 49 cultures as described by [12]. Briefly, ACN/water (60/40, 50 mL *v/v*) were added to 10 g of macerated mycelia containing agar in a conical flask (250 mL), which was shaken for 1 h. Thereafter, the mixture was passed through a Whatman #4 filter paper (Merck, Darmstadt, Germany), and the pH of the filtrate was adjusted to 6.2 ± 0.3 using 1 M sulphuric acid. The filtrate was emptied into a 250 mL separation funnel (and extracted (×3) with DCM (25 mL). Acetonitrile (25 mL) was added to the extract, in order to eliminate moisture, the mixture was passed through sodium sulphate anhydrous (Merck, Darmstadt, Germany) and dried with nitrogen gas. Other steps were followed as previously demonstrated for the *Aspergillus* and *Penicillium* extracts.

### 4.6. Instrumentation Identification and Quantification of Aspergillus Metabolites

The capability of *Aspergillus* in producing mycotoxins and other secondary metabolites was established using an Acquity UPLC (Waters, Milford, MA, USA) attached to a XEVO TQ-S triple quadrupole UPLC/MS/MS (Waters, Milford, MA, USA). The data acquisition and processing were done using the MassLynx™ V. 4.1 and QuanLynx® V. 4.1 software (Manchester, UK). The analytes chromatographic separation was carried out with the HSS T3 (100 × 2.1 mm, 1.8 µm) column (Waters, Zellik, Belgium) with a BEH C18 guard column (2.1 × 5 mm, 1.7 µm) (Waters, Zellik, Belgium). Mobile phase A and B were pumped at a flow rate of 0.4 mL/min. The run time of each analysis was 32 min, injection volume of the sample was 5 μL and the pressure ranged between 0 and 15,000 psi. Ionization was performed in the ESI+ (positive electrospray ionization) mode and data was acquired in the MRM (multiple reaction monitoring) mode. For the investigation of the secondary metabolites, where no reference standards were available, accurate masses were determined using the LC-SYNAPT G-2 high-resolution mass spectrophotometer (Waters, Zellik, Belgium). More information is detailed in Okoth et al. [23]. For each of the 34-targeted *Aspergillus* analytes, two-product ion transitions were selected and their collision energies optimized. For the quantification of AFB1, AFB2, AFG1, AFG2, OTA, STE, DAS, T-2, FB_3_, ZEN, DON, FB_1_, FB_2_, 3-AcDON, 15-AcDON, NEO, NIV, HT-2, and FUS-X, matrix-matched calibration curves were used. Additional details on the *Aspergillus* metabolites transitions are in Table 3.

Instrumental settings included: capillary voltage of 3 kV, desolvation temperatures of 500 °C, source offset voltage of 50 V, cone voltage of 10 V, cone and desolvation gas flows of 150 and 1000 L/h. The European Commission (EC) no. 2006 [41] criteria were met in order to establish the identities of the targeted metabolites. This method is validated for most of the metabolites based on EC recommendations [42], and further information on this method is given by [23].

### 4.7. Detection and Quantification of Penicillium and Fusarium Metabolites

An Acquity UPLC (Waters, Zellik, Belgium) equipment coupled to a Quattro Premier XE LC/MS/MS (Waters, Zellik, Belgium) was used to detect and quantify both *Penicillium* and *Fusarium* metabolites. The spectrometric and chromatographic conditions used were similar to those described by Ediage et al. [43] and further gradation details of the of the metabolites are reported by De Boevre et al. [44]. Also, the EC [41] guideline was followed for the identification of targeted metabolites. 

### 4.8. Statistical Analysis

The R software V. 2.153 (R core Team, 2014, R Foundation for Statistical Computing, Vienna, Austria) was employed. To assemble the isolates based on their secondary metabolites production data and to cluster the metabolites, a hierarchical grouping, established upon the simple matching distance principle wherein variation between binary sample sets are measured was utilized. With the use of a probabilistic model [45], pairwise associations amongst the isolates based on their metabolite profile was done upon which they were categorized as positive, random, or negative. 

## Figures and Tables

**Table 1:** Production of mycotoxins by *Aspergillus*, *Penicillium*, and *Fusarium* spp. isolated from lactic acid fermented products produced in Nigeria and South Africa.

Isolated Species	Accession No.	*Ogi*			*Ogi baba*			*Umqombothi*			*Mahewu*		
		No. of Iso. Spp. (%)	No. of Tox. Spp.	Toxin Produced (Range: µg/kg)	No. of Iso. Spp. (%)	No. of Tox. Spp.	Toxin Produced (Range: µg/kg)	No. of Iso. Spp. (%)	No. of Tox. Spp.	Toxin Produced (Range: µg/kg)	No. of Iso. Spp. (%)	No. of Tox. Spp.	Toxin Produced (Range: µg/kg)
*Aspergillus* Species													
*A. amstelodami*	AY373885.1	1 (2)	1	STE (371)	-	-	-	-	-	-	-	-	-
*A. clavatus*	KU052566	2 (5)	-	-	2 (13)	-	-	-	-	-	3 (14)	-	-
*A. flavus*	KR611584	12 (29)	7, 3	AFB_1_ (109–231), STE (434–600)	6 (38)	4, 3	AFB_1_ (217–1556), STE (49–701)	6 (26)	6, 3	AFB_1_ (127–1117), STE (4–32)	7 (32)	4, 1	AFB_1_ (69–1931), STE (119)
*A. fumigatus*	KX215145.1	4 (10)	-	-	-	-	-	2 (9)	-	-	3 (14)	-	-
*A. minisclerotigenes*	JF412778	-	-	-	1 (6)	-	-	2 (9)	1	AFB_1_ (96) AFB_2_ (106)	-	-	-
*A. niger*	KX215115.1	7 (17)	7	OTA (197–1302)	1 (6)	1	OTA (411)	3 (13)	2	OTA (28–55)	3 (14)	2	OTA (89–212)
*A. parasiticus*	DQ467988.1	7 (17)	5, 5, 4, 3	AFB_1_ (89–1018), AFB_2_ (118–324), AFG_1_ (95–158), AFG_2_ (978–664)	2 (13)	2, 2, 1, 1	AFB_1_ (721–1030), AFB_2_ (185–498), AFG_1_ (48), AFG_2_ (34)	3 (13)	2, 2, 1	AFB_1_ (172–3602), AFB_2_ (288–566), AFG_1_ (322)	3 (14)	2, 2, 1	AFB_1_ (109–117), AFB_2_ (58–192), AFG_1_ (109)
*A. ruber*	KX215127.1	1 (2)	-	-	-	-	-	-	-	-	-	-	-
*A. spp*	KX215117.1	-	-	-	1 (13)	-	-	-	-	-	-	-	-
*A. sclerotiorum*	KT717312	-	-	-	-	-	-	2 (9)	-	-	-	-	-
*A. sydowii*	KR611596	1 (2)	1	STE (91)	1 (13)	1	STE (433)	2 (9)	1	STE (53)	-	-	-
*A. tritici*	KP780810	2 (5)	-	-	1 (13)	-	-	2 (9)	-	-	-	-	-
*A. tubingensis*	KT717311	-	-	-	-	-	-	-	-	-	1 (5)	-	-
*A. ustus*	HQ607918.1	-	-	-	-	-	-	-	-	-	-	-	-
*A. versicolor*	LC105698	4 (10)	2	STE (97–477)	1 (13)	1	STE (422)	1 (4)	1	STE (54)	2 (9)	-	-
*Penicillium* species													
*P. chrysogenum*	KP836338	5 (39)	1	ROQ C (22)	-	-	-	3 (38)	1	ROQ C (278)	2 (4)	-	-
*P. expansum*	AY818338	-	-	-	2 (33)	-	-	-	-	-	-	-	-
*P. aethiopicum*	KX215125.1	-	-	-	-	-	-	3 (38)	-	-	-	-	-
*P. camemberti*	KT355012	2 (15)	-	-	-	-	-	-	-	-	-	-	-
*P. citrinum*	KX215134.1	3 (23)	-	-	1 (17)	-	-	-	-	-	-	-	-
*P. crustosum*	KT735107.1	2 (15)	-	-	-	-	-	2 (25)	-	-	3 (6)	-	-
*P. raistrickii*	KX215126.1	-	-	-	2 (33)	-	-	-	-	-	-	-	-
*P. mallochi*	KX215135.1	-	-	-	1 (17)	-	-	-	-	-	-	-	-
*Fusarium* species													
*F. chlamydosporum*	KX215137.1	1 (43)	-	-	-	-	-	-	-	-	-	-	-
*F. fujikuroi*	KX215132.1	-	-	-	1 (33)	-	-	-	-	-	-	-	-
*F. sporotrichioides*	AF404149.1	2 (29)	1, 1, 2	HT-2 (127), DAS, NEO	-	-	-	-	-	-	-	-	-
*Fusarium* sp.	KP003945	-	-	-	2 (67)	2, 2, 1	FB_3_ (79), DON (27-300), 3-AcDON	-	-	-	2 (40)	2	ZEN (180–309)
*F. verticillioides*	KX215138.1	4 (57)	4	FB_1_ (77–218)	-	-	-	3 (100)	3	FB_1_ (92–109)	3 (60)	3, 1	FB_2_ (103–195) FB_3_ (88)

No.: number; Iso.: isolated; Tox.: toxigenic; Only *Aspergillus* metabolites with available standards for quantification (AFB_1_, AFB_2_, STE, OTA, AFG_1_, AFG_2_,) are presented in Table 1; Aflatoxin: B_1_ (AFB_1_), B_2_ (AFB_2_),G_1_ (AFG_1_), G_2_ (AFG_2_), neosolaniol (NEO), deoxynivalenol (DON), sterigmatocystin (STE), diacetoxyscirpenol (DAS), (T-2), fumonisin B_3_ (FB_3_), zearalenone (ZEN), fumonisin B_2_ (FB_2_), 3-acetyldeoxynivalenol (3-AcDON), ochratoxin A (OTA), nivalenol (NIV), fumonisin B_1_ (FB_1_), HT-2 toxin (HT-2), T-2 toxin, and fusarenon-X (FUS-X); Given numbers in the mid-column of each product indicate the number of toxigenic species among the isolated species. The mycotoxin(s) produced by this toxigenic species and range are indicted in the next column, e.g., for *ogi*, 5 of 7, 5 of 7, 4 of 7, and 3 of 7 *A. parasiticus* isolate found in *ogi* were toxigenic and they respectively produced AFB_1_, (range: 89–1018 µg/kg), AFB_2_ (118–324 µg/kg), AFG_1_ (95–158 µg/kg), and AFG_2_ (978–664 µg/kg).

**Table 2:** Production of mycotoxins by *Aspergillus*, *Penicillium*, and *Fusarium* spp. isolated from alkaline fermented products produced in Nigeria and South Africa.

Isolated Species	Accession No.	*Ugba*			*Ogiri*			*Iru*		
		No. of Iso. Spp. (%)	No. of Tox. Spp.	Toxin Produced (Range: µg/kg)	No. of Iso. Spp. (%)	No. of Tox. Spp.	Toxin Produced (Range: µg/kg)	No. of Iso. Spp. (%)	No. of Tox. Spp.	Toxin Produced (Range: µg/kg)
*A. amstelodami*	AY373885.1	-	-	-	-	-	-	3 (5)	-	-
*A. candidus*	KT223337	2 (7)	-	-	-	-	-	3 (5)	-	-
*A. clavatus*	KUO52566	-	-	-	1 (2)	-	-	3 (5)	-	-
*A. flavus*	KR611584	12 (39)	10, 12	AFB_1_ (27–1889), STE (28–325)	15 (36)	9, 9	AFB_1_ (96–7406), STE (94–736)	22 (34)	16, 4	AFB_1_ (82–1723), STE (77–128)
*A. fumigatus*	KU684451	3 (8)	-	-	6 (14)	-	-	10 (15)	-	-
*A. minisclerotigenes*	JF412778	-	-	-	3 (7)	1	AFB_1_ (242)	-	-	-
*A. niger*	KX215111.1	3 (8)	3	OTA (76–1265)	4 (10)	2	OTA (118–229)	3 (5)	1	OTA (78)
*A. oryzae*	KX215113.1	-	-	-	2 (5)	-	-	-	-	-
*A. parasiticus*	DQ467988.1	4 (13)	4, 4, 1	AFB_1_ (391–1132), AFB_2_ (37–504), AFG_1_ (46)	6 (14)	5, 5, 1, 1	AFB_1_ (120–1470), AFB_2_ (83–323), AFG_1_ (69), AFG_2_ (97)	11 (17)	8, 3, 2, 2	AFB_1_ (206–445), AFB_2_ (51–340), AFG_1_ (36–59), AFG_2_ (49–71)
*A. ruber*	KX215127.1	-	-	-	-	-	-	1 (2)	-	-
*A. spp*	KX215117.1	1 (3)	-	-	-	-	-	-	-	-
*A. sclerotiorum*	KT717312	3 (10)	1	OTA (161)	-	-	-	2 (3)	-	-
*A. sydowii*	KX215130.1	-	-	-	-	-	-	2 (3)	-	-
*A. tritici*	KX215119.1	-	-	-	-	-	-	-	-	-
*A. tubingensis*	KT717311	2 (7)	-	-	-	-	-	2 (3)	-	-
*A. ustus*	HQ607918.1	-	-		3 (7)	-	-	-	-	-
*A. versicolor*	LC105698	1 (3)	1	STE (101)	2 (5)	2	STE (89–500)	3 (5)	1	STE (89)
*P. chrysogenum*	KX215133.1	4 (29)	4	ROQ C (360–1260)	6 (30)	-	-	9 (30)	6	ROQ C (13–57)
*P. expansum*	AY818338	1 (7)	-	-	-	-	-	2 (7)	-	-
*P. polonicum*	KX215146.1	-	-	-	-	-	-	1 (3)	-	-
*P. lanosocoeruleum*	JX997110	-	-	-	-	-	-	2 (7)	-	-
*P. aethiopicum*	KX215125.1	2 (14)	-	-	2 (10)	-	-	-	-	-
*P. camemberti*	KT355012	-	-	-	-	-	-	1 (3)	-	-
*p. citrinum*	KX215122.1	-			2 (10)	-	-	2 (7)		-
*p. verrucosum*	KM115130	2 (14)	1	OTA (15)	1 (5)	1	OTA (19)	3 (10)	2	OTA (18–32)
*p. crustosum*	KT735107.1	2 (14)	-	-	3 (15)	-	-	-	-	-
*P. flavigenum*	LN809058	-	-	-	2 (10)	-	-	-	-	-
*P. raistrickii*	KX215126.1	-	-	-	4 (20)	-	-	3 (10)	-	-
*P. glabrum*	JN887323.1	2 (14)	-	-	-	-	-	1 (3)	-	-
*P. rubens*	LC105692	-	-	-	-	-	-	2 (7)	-	-
*P. steckii*	KX215128.1	1 (7)	-	-	-	-	-	1 (3)	-	-
*P. mallochi*	KX215135.1	-	-	-	-	-	-	3 (10)	-	-
*F. andiyazi*	KX215140.1	2 (25)	-	-	-	-	-	-	-	-
*F. chlamydosporum*	KP769538.1	-	-	-	-	-	-	4 (32)	1	DAS
*F. fujikuroi*	KT192328	-	-	-	1 (10)	-	-	-	-	-
*F. proliferatum*	KP773280	2 (25)	-	-	2 (20)	-	-	2 (16)	-	-
*F. sporotrichioides*	AF404149.1	1 (13)	1	NEO	2 (20)	2, 2	T-2 (134), DAS	2 (8)	2, 2, 2	T-2 (139–1749), DAS, FUS-X
*Fusarium* sp.	JQ350882	1 (13)	1, 1, 1	DON (870), ZEN (197), 3-AcDON	-	-	-	1 (8)	1, 1	ZEN (139)
*F. verticillioides*	KX215131.1	2 (38)	1, 1, 1	FB_1_ (81), FB_2_ (63), FB_3_ (205)	5 (50)	1, 3	FB_2_ (234), FB_3_ (79–148)	4 (31)	-	-

No.: number; Iso.: isolated; Tox.: toxigenic; Only *Aspergillus* metabolites with available standards for quantification (AFB_1_, AFB_2_, STE, OTA, AFG_1_, AFG_2_) are presented in Table 2; Given numbers in the mid-column of each product indicate the number of toxigenic species among the isolated species. The mycotoxin(s) produced by this toxigenic species and range are indicted in the next column, e.g., for *ugba*, 10 of 12, and 12 of 12 of the *A. flavus* isolate found in *ugba* were toxigenic and they respectively produced AFB_1_ (27–1889 µg/kg) and STE (28–325 µg/kg).

**Table 3:** Mass spectrometric parameters for different *Aspergillus* metabolites.

Component	Abbreviation	Precursor Ion (m/z)	Product Ion (m/z)	Cone Voltage (V)	Collision Energy (eV)	Expected Retention Time (min)
* Kojic acid	KA	143.1	69.1 125.1	35	30	3.70
* Methylisocoumarin	ME-ISOC	307.1	149.1 247.1	35	35	8.68
Aflatoxin G_2_	AFG_2_	331.1	245.1 313.1	25	18	8.95
Aflatoxin G_1_	AFG_1_	329.1	243.1 311.0	35	25	9.00
Aflatoxin B_2_	AFB_2_	315.1	259.1 286.9	25	35	9.43
* Speradine A	SPRE	367.2	160.1 266.1	35	35	9.72
Aflatoxin B_1_	AFB_1_	313.1	270.1 285.1	70	35	9.92
* Sterigmatocystin Analogue	STE-A	325.1	281.1 310.1	35	34	9.95
* Ochratoxin A	OTA	214.0	142.0 152.0	51	25	9.96
* Oxy-o-methyl Sterigmatocystin	OxyHOMST	371.1	282.1 315.1	35	35	10.10
* Dihydroxyl-o-methyl STE	DHoxyHOMST	373.1	322.1 355.1	35	35	10.32
* Aflavarin	AFV	455.2	379.2 413.2	35	40	10.35
* Aspertoxin 1	ASPT	355.1	322.1 340.1	35	30	10.51
* Aspertoxin 2	ASPT-2	355.1	322.1 340.1	35	40	10.53
* Aspergillic acid	AA	225.2	165.1 207.2	35	40	10.70
* Versiconol	VOH	361.1	285.1 325.1	35	35	11.13
* Aflavarin-Analog 2	AFV-2	425.1	334.1 383.1	35	35	11.24
* Dihydro-o-methyl Sterigmatocystin	DHOMST	341.1	285.1 326.1	35	35	11.49
* Versiconal Hemiacetal Acetate	VHA	401.1	283.1 307.1	35	35	11.52
* Flavacol	FLV	209.2	123.1 137.1	35	40	11.78
* Dehydro-Aflavanine	DH-AF	438.3	285.2 402.3	35	40	12.17
* O-methyl Sterigmatocystin	OMST	339.1	306.1 324.1	35	35	12.69
* Aflavarin-Analog 1	AFV-1	439.1	365.1 397.1	35	35	12.93
* Noranthrone	NORA	357.2	245.1 273.1	35	35	13.02
* Aflatrem	AFTR	502.3	156.1 198.1	35	40	13.17
* Paspalinine	PASL	434.2	130.1 376.2	35	40	13.78
Sterigmatocystin	STE	325.0	281.1 310.1	35	34	14.28
* Cyclopiazonic acid	CPA	337.2	140.1 196.1	35	40	14.29
* Hydroxyneoaspergillic acid	OH-AA	241.2	137.1 163.1	35	40	14.31
* ß-Cyclopiazonic acid	ß-CPA	339.2	154.1 198.1	35	40	14.75
* Dytryptohenaline	DYT	693.3	318.2 346.2	35	40	14.80
* Paspaline	PAS	422.3	158.1 386.3	35	35	15.04
* Leporin C	LEO-C	336.2	200.1 214.1	35	40	17.40
* Aflavinine	AFN	406.3	180.1 224.3	35	45	19.38

* Standard not available for quantitative determination.

## Supplementary Materials

The following are available online at http://www.mdpi.com/2072-6651/11/2/85/s1. Supplementary data on *Aspergillus* species isolated from fermented foods and beverage from Nigeria and South Africa as shown in Figure 1 and Figure 2.
