# Supplementary material for: Investigation of the Metabolic Profile and Toxigenic Variability of Fungal Species Occurring in Fermented Foods and Beverage from Nigeria and South Africa Using UPLC-MS/MS
Source: Toxins (Basel). 2019 Feb 1;11(2):85. doi: 10.3390/toxins11020085 (PMC6409632; doi:10.3390/toxins11020085)
Supplement: Supplementary file 1 [file toxins-11-00085-s001.pdf]

Supplementary Materials: Investigation of the Metabolic Profile and Toxigenic Variability of Fungal Species Occurring in Fermented Foods and Beverage from Nigeria and South Africa Using UPLC-MS/MS

Ifeoluwa Adekoya, Patrick Njobeh, Adewale Obadina, Sofie Landschoot, Kris Audenaert, Sheila Okoth, Marthe De Boevre, and Sarah De Saeger

Supplementary data: *Aspergillus species* in fermented foods as shown in Figures 1 and 2.

**Nigerian Fermented Food Samples**	**Isolates species as shown in Figure 1 and corresponding numbers**	
Ogiri	*A.flavus*	1
Ogiri	*A.flavus*	2
Ogiri	*A.flavus*	3
Ogiri	*A.flavus*	4
Ogiri	*A.flavus*	5
Ogiri	*A.flavus*	6
Ogiri	*A.flavus*	7
Ogiri	*A. fumigatus*	8
Ogiri	*A. fumigatus*	9
Ogiri	*A. fumigatus*	10
Ogiri	*A. minisclerotigenes*	11
Ogiri	*A. parasiticus*	12
Ogiri	*A. parasiticus*	13
Ogiri	*A. parasiticus*	14
Ogiri	*A. niger*	15
Ogiri	*A. niger*	16
Ogiri	*A. ustus*	17
Ogiri	*A. versicolor*	18
Ogiri	*A. clavatus*	19
Iru	*A. candidus*	20
Iru	*A. clavatus*	21
Iru	*A.flavus*	22
Iru	*A.flavus*	23
Iru	*A.flavus*	24
Iru	*A.flavus*	25
Iru	*A.flavus*	26
Iru	*A.flavus*	27
Iru	*A.flavus*	28
Iru	*A.flavus*	29
Iru	*A.flavus*	30
Iru	*A.flavus*	31
Iru	*A.fumigatus*	32
Iru	*A.fumigatus*	33
Iru	*A.fumigatus*	34

Iru	*A.fumigatus*	35
Iru	*A.fumigatus*	36
Iru	*A.fumigatus*	37
Iru	*A.niger*	38
Iru	*A.parasiticus*	39
Iru	*A.parasiticus*	40
Iru	*A.parasiticus*	41
Iru	*A.parasiticus*	42
Iru	*A.parasiticus*	43
Iru	*A.ruber*	44
Iru	*A.sclerotium*	45
Iru	*A.versicolor*	46
Iru	*A.versicolor*	47
Ugba	*A.flavus*	48
Ugba	*A.flavus*	49
Ugba	*A.flavus*	50
Ugba	*A.flavus*	51
Ugba	*A.flavus*	52
Ugba	*A.flavus*	53
Ugba	*A.flavus*	54
Ugba	*A.fumigatus*	55
Ugba	*A.niger*	56
Ugba	*A.parasiticus*	57
Ugba	*A.parasiticus*	58
Ugba	*A.spp*	59
Ugba	*A.sclerotium*	60
Ugba	*A.tubingensis*	61
Ugba	*A.tubingensis*	62
Ugba	*A.versicolor*	63
Ogi	*A.amstelodami*	64
Ogi	*A.flavus*	65
Ogi	*A.flavus*	66
Ogi	*A.flavus*	67
Ogi	*A.flavus*	68
Ogi	*A.flavus*	69
Ogi	*A.flavus*	70
Ogi	*A.fumigatus*	71
Ogi	*A.fumigatus*	72
Ogi	*A.fumigatus*	73
Ogi	*A.niger*	74
Ogi	*A.niger*	75
Ogi	*A.niger*	76
Ogi	*A.niger*	77
Ogi	*A.niger*	78
Ogi	*A.parasiticus*	79

Ogi	*A.parasiticus*	80
Ogi	*A.parasiticus*	81
Ogi	*A.parasiticus*	82
Ogi	*A.parasiticus*	83
Ogi	*A.ruber*	84
Ogi	*A.versicolor*	85
Ogi	*A.versicolor*	86
Ogi Baba	*A.clavatus*	87
Ogi Baba	*A.clavatus*	88
Ogi Baba	*A.flavus*	89
Ogi Baba	*A.flavus*	90
Ogi Baba	*A.flavus*	91
Ogi Baba	*A.flavus*	92
Ogi Baba	*A.flavus*	93
Ogi Baba	*A.flavus*	94
Ogi Baba	*A.minisclerotigenes*	95
Ogi Baba	*A.niger*	96
Ogi Baba	*A.parasiticus*	97
Ogi Baba	*A.parasiticus*	98
Ogi Baba	*A.spp*	99
Ogi Baba	*A.sydowii*	100
Ogi Baba	*A.tritci*	101
Ogi Baba	*A.versicolor*	102

**South African Fermented Food Samples**	**Isolates species as shown in Figure 2 and corresponding numbers**	
Ogiri	*A.flavus*	1
Ogiri	*A.flavus*	2
Ogiri	*A.flavus*	3
Ogiri	*A.flavus*	4
Ogiri	*A.flavus*	5
Ogiri	*A.flavus*	6
Ogiri	*A.flavus*	7
Ogiri	*A.flavus*	8
Ogiri	*A.fumigatus*	9
Ogiri	*A.fumigatus*	10
Ogiri	*A.fumigatus*	11
Ogiri	*A.minisclerotigenes*	12
Ogiri	*A.minisclerotigenes*	13
Ogiri	*A.niger*	14
Ogiri	*A.niger*	15
Ogiri	*A.oryzae*	16
Ogiri	*A.oryzae*	17
Ogiri	*A.parasiticus*	18
Ogiri	*A.parasiticus*	19
Ogiri	*A.parasiticus*	20

Ogiri	*A.ustus*	21
Ogiri	*A.ustus*	22
Ogiri	*A.versicolor*	23
Iru	*A.amstelodami*	24
Iru	*A.amstelodami*	25
Iru	*A.candidus*	26
Iru	*A.candidus*	27
Iru	*A.candidus*	28
Iru	*A.candidus*	29
Iru	*A.flavus*	30
Iru	*A.flavus*	31
Iru	*A.flavus*	32
Iru	*A.flavus*	33
Iru	*A.flavus*	34
Iru	*A.flavus*	35
Iru	*A.flavus*	36
Iru	*A.flavus*	37
Iru	*A.flavus*	38
Iru	*A.flavus*	39
Iru	*A.flavus*	40
Iru	*A.flavus*	41
Iru	*A.fumigatus*	42
Iru	*A.fumigatus*	43
Iru	*A.fumigatus*	44
Iru	*A.fumigatus*	45
Iru	*A.niger*	46
Iru	*A.niger*	47
Iru	*A.parasiticus*	48
Iru	*A.parasiticus*	49
Iru	*A.parasiticus*	50
Iru	*A.parasiticus*	51
Iru	*A.parasiticus*	52
Iru	*A.parasiticus*	53
Iru	*A.sclerotiorum*	54
Iru	*A.sclerotiorum*	55
Iru	*A.sydowii*	56
Iru	*A.sydowii*	57
Iru	*A.tubingensis*	58
Iru	*A.tubingensis*	59
Iru	*A.versicolor*	60
Ugba	*A.candidus*	61
Ugba	*A.candidus*	62
Ugba	*A.flavus*	63
Ugba	*A.flavus*	64
Ugba	*A.flavus*	65

Ugba	*A.flavus*	66
Ugba	*A.flavus*	67
Ugba	*A.fumigatus*	68
Ugba	*A.fumigatus*	69
Ugba	*A.niger*	70
Ugba	*A.niger*	71
Ugba	*A.parasiticus*	72
Ugba	*A. parasiticus*	73
Ugba	*A.sclerotiorum*	74
Ugba	*A.sclerotiorum*	75
Ogi	*A.clavatus*	76
Ogi	*A.clavatus*	77
Ogi	*A.flavus*	78
Ogi	*A.flavus*	79
Ogi	*A.flavus*	80
Ogi	*A.flavus*	81
Ogi	*A.flavus*	82
Ogi	*A.flavus*	83
Ogi	*A.fumigatus*	84
Ogi	*A.niger*	85
Ogi	*A.niger*	86
Ogi	*A.parasiticus*	87
Ogi	*A.parasiticus*	88
Ogi	*A.sydowii*	89
Ogi	*A.tritici*	90
Ogi	*A.tritici*	91
Ogi	*A.versicolor*	92
Ogi	*A.versicolor*	93
Mahewu	*A.clavatus*	94
Mahewu	*A.clavatus*	95
Mahewu	*A.clavatus*	96
Mahewu	*A.flavus*	97
Mahewu	*A.flavus*	98
Mahewu	*A.flavus*	99
Mahewu	*A.flavus*	100
Mahewu	*A.flavus*	101
Mahewu	*A.flavus*	102
Mahewu	*A.flavus*	103
Mahewu	*A.fumigatus*	104
Mahewu	*A.fumigatus*	105
Mahewu	*A.fumigatus*	106
Mahewu	*A.niger*	107
Mahewu	*A.niger*	108
Mahewu	*A.niger*	109
Mahewu	*A.parasiticus*	110
Mahewu	*A.parasiticus*	111

Mahewu	*A.parasiticus*	112
Mahewu	*A.tubingensis*	113
Mahewu	*A.versicolor*	114
Mahewu	*A.versicolor*	115
Umqombothi	*A.flavus*	116
Umqombothi	*A.flavus*	117
Umqombothi	*A.flavus*	118
Umqombothi	*A.flavus*	119
Umqombothi	*A.flavus*	120
Umqombothi	*A.flavus*	121
Umqombothi	*A.fumigatus*	122
Umqombothi	*A.fumigatus*	123
Umqombothi	*A.minisclerotigenes*	124
Umqombothi	*A.minisclerotigenes*	125
Umqombothi	*A.niger*	126
Umqombothi	*A.niger*	127
Umqombothi	*A.niger*	128
Umqombothi	*A.parasiticus*	129
Umqombothi	*A.parasiticus*	130
Umqombothi	*A.parasiticus*	131
Umqombothi	*A.sclerotiorum*	132
Umqombothi	*A.sclerotiorum*	133
Umqombothi	*A.sydowii*	134
Umqombothi	*A.sydowii*	135
Umqombothi	*A.tritici*	136
Umqombothi	*A.tritici*	137
Umqombothi	*A.versicolor*	138
